# Risk-based guidance for choosing fecal immunochemical test or colonoscopy in colorectal cancer screening: a modeling study

**DOI:** 10.1093/aje/kwaf214

**Published:** 2025-09-30

**Authors:** Luuk A van Duuren, Jean-Luc Bulliard, Matthias Harlass, Ekaterina Plys, Douglas A Corley, Florian Froehlich, Kevin Selby, Iris Lansdorp-Vogelaar

**Affiliations:** Department of Public Health, Erasmus MC, University Medical Center, Rotterdam, The Netherlands; Unisanté, Center for Primary Care and Public Health, University of Lausanne, Lausanne, Switzerland; Unisanté, Center for Primary Care and Public Health, University of Lausanne, Lausanne, Switzerland; Department of Public Health, Erasmus MC, University Medical Center, Rotterdam, The Netherlands; Unisanté, Center for Primary Care and Public Health, University of Lausanne, Lausanne, Switzerland; Division of Research, Kaiser Permanente Northern California, Oakland, CA, United States; Department of Gastroenterology, University of Basel, Basel, Switzerland; Unisanté, Center for Primary Care and Public Health, University of Lausanne, Lausanne, Switzerland; Department of Public Health, Erasmus MC, University Medical Center, Rotterdam, The Netherlands

**Keywords:** personalized screening, Qcancer, screening recommendations, stool test, microsimulation

## Abstract

In colorectal cancer (CRC) screening settings offering both colonoscopy and fecal immunochemical test (FIT), guidance on who should get colonoscopy could optimize resource use. This study aimed to identify efficient guidance strategies, maximizing quality-adjusted lifeyears (QALYs) gained for given colonoscopy demand. Using the MISCAN-Colon microsimulation model for Switzerland, we evaluated 3 strategy types: age-based, starting biennial FIT and switching to 10-yearly colonoscopy at a certain age; risk score–based, where only individuals with high CRC risk scores undergo colonoscopy; FIT-based, switching to colonoscopy after a quantitative FIT result just below the positivity cut-off and, in some strategies, also at a certain age. Reference strategies included (1) colonoscopy only and (2) equal proportions of individuals choosing FIT or colonoscopy at age 50. Age- and risk score–based strategies with switches or risk assessments at ages 54, 64, or 74 were efficient. Compared to the reference strategies, QALYs gained could increase by (1) 10.0% or (2) 6.7% without increasing colonoscopy demand. The FIT-based switching strategies were not efficient. Therefore, screening programs like those in Switzerland and the United States can improve efficiency by guiding individuals toward FIT or colonoscopy simply based on age. More complex approaches using prior FITs or risk scores would not outperform age-based approaches.

## Introduction

Colorectal cancer (CRC) is a common and deadly disease with nearly 2 million cases and 1 million deaths worldwide in 2020.^1^ Fortunately, screening effectively reduces CRC incidence and mortality,^2^^,^^3^ and CRC screening programs have been implemented in many countries.^4^^-^^6^ The most commonly used screening tests are colonoscopy and fecal immunochemical test (FIT). Colonoscopy is more sensitive than FIT, especially for precancerous lesions. However, it also requires bowel preparation and carries the risk of colonic perforations.^7^ Further, it occupies the time of trained specialists, has a substantial carbon footprint, and is associated with much higher costs.^8^^-^^10^ Thus, colonoscopy resources could be reserved for individuals at higher risk of CRC, especially if colonoscopy capacity is restricted.

Participants can choose between FIT and colonoscopy for CRC screening in settings such as the United States, Germany, and most cantons in Switzerland.^5^^,^^11^^,^^12^ Some guidelines suggest 1 of the 2 tests based on age or other risk factors. In Germany, guidelines suggest colonoscopy above age 55, but participants may also choose FIT. Similarly, colonoscopy is suggested in the canton of Vaud, Switzerland, for previously unscreened individuals aged 65 and older. At the individual level, these approaches facilitate a straightforward, informed screening choice, weighing the potential harms of colonoscopy against the lower sensitivity of FIT. At a population level, this may lead to a more efficient allocation of scarce colonoscopy resources.

Previous modeling studies and a scoping review on risk-stratified CRC screening concluded that such hybrid screening strategies using both FIT and colonoscopy could improve efficiency.^13^^-^^15^ However, the optimal age to switch from FIT to colonoscopy has not been studied, and it is uncertain whether information on CRC risk factors such as body mass index or smoking may enhance these suggestions. A recent systematic review showed that the detection of advanced neoplasia increases with fecal hemoglobin levels in prior negative FIT tests.^16^ Thus, these hemoglobin levels could also inform who should switch from FIT to colonoscopy.

Recently, the PREcision ScreENing pilot randomized controlled Trial (PRESENT) in Switzerland experimented with patient-level guidance toward FIT or colonoscopy based on predicted individual CRC risk.^17^ The trial used the QCancer-colorectal risk tool to estimate 15-year CRC risk based on factors like age, sex, body mass index (BMI), alcohol, and smoking status,^18^^,^^19^ which demonstrated the highest discriminatory performance in a comparative external validation study using UK Biobank data.^20^ In the PRESENT intervention arm, low-risk participants (CRC risk <3%) received a brochure suggesting FIT, high-risk participants (≥6%) were suggested colonoscopy, and those at moderate risk (3%-6%) received no specific suggestion. The trial found that low-risk participants who received a tailored brochure were more likely to choose FIT, without impacting overall screening uptake.^21^

The aims of this study were to estimate the long-term, population-level impact of guidance for colonoscopy or FIT, and to identify the strategies achieving highest benefits for given colonoscopy requirements. We evaluated 3 types of guidance strategies where individuals underwent FIT or colonoscopy based on (1) age, (2) the QCancer risk score like in the PRESENT trial, and (3) quantitative FIT results just below the positivity cut-off. We then assessed whether such strategies could maintain the current screening benefits of screening while reducing colonoscopy requirements.

## Methods

We used the MIcrosimulation SCreening ANalysis-Colon (MISCAN-Colon) model to evaluate 3 types of risk-based strategies to guide choices between FIT or colonoscopy: (1) age-based, (2) risk score–based using the QCancer risk score, and (3) FIT-based using the most recent FIT result. For each strategy, the target population for screening was stratified into high and low risk of CRC. Individuals at high risk underwent 10-yearly colonoscopy and those at low risk biennial FIT. During their lifetime, individuals could be reclassified from low to high risk, prompting a switch to colonoscopy screening. These strategies were compared to 3 reference strategies. We identified efficient strategies that minimized the number of colonoscopies required for screening and surveillance, and maximized quality-adjusted lifeyears (QALYs) gained at a population level. The Swiss population was used as a case study.

### MISCAN-Colon microsimulation model

MISCAN-Colon is a well-established microsimulation model implemented in Python 3. It has been extensively validated against randomized controlled trials^22^^,^^23^ and has informed CRC screening guidelines in multiple countries.^24^^-^^26^ The structure and underlying assumptions of the model have been described previously.^25^^,^^27^ In short, this semi-Markov model simulates a population from birth to death at the individual level. Death from other causes than CRC is scheduled at the start of an individual’s simulation. Some individuals develop adenomas during their lifetime, which may grow and progress to CRC following the adenoma-carcinoma pathway. Once a cancer shows clinical symptoms, it is treated and a time to death is determined based on the individual’s age, cancer stage, and localization.

If CRC screening is added to the simulation, individuals follow a predefined screening schedule, altering some simulated life histories. The benefits and harms of strategies can be compared by simulating various screening strategies in identical populations.

This study used a version of MISCAN-Colon that was previously adapted to the Swiss population based on data from the Swiss Federal Statistics Office and the Swiss cancer registries (Appendix S1). The QCancer risk prediction tool was previously incorporated using data from the population-based Swiss Health Survey.^28^^,^^29^

### Screening strategies

For age-based, risk score–based and FIT-based guidance, we evaluated multiple strategies. Each strategy used different criteria to stratify between high- and low-risk individuals (Table 1). Simulated individuals at low CRC risk underwent biennial FIT (cut-off 15 μg/g) and those at high risk 10-yearly colonoscopy. In the base case, screening always started at age 50 and ended at age 74.

**Table 1:** Overview of screening strategies per risk classification type.

**Classification approach**	**High-risk criterion**	**Possible values**	**Possible transition moments**	**Number of strategies**
Age-based	Reaching the transition age.	52, 54, …, 72, 74	At the transition age only	12
Risk score–based (see Appendix S2)	Reaching the specified proportion of top QCancer-predicted risk scores among the assessed population.	Possible proportions: 10%, 20% …, 90%	At risk assessments	1575
FIT-based	An elevated but negative FIT result (and in some strategies, reaching the transition age).	5-15 μg/g, 10-15 μg/g (and 54, 56, …, 72, 74)	After each FIT (or at the transition age)	24
Reference strategies	None	FIT only, COL only, and 50/50^a^	None	3

a Scenario in which 50% of individuals does biennial FIT and 50% does 10-yearly colonoscopy from the age of 50.

#### Age-based strategies

In age-based strategies, participants were reclassified from low to high risk at a certain *transition age*. We simulated transition ages between 52 and 74, in steps of 2 years. For example, the age-based strategy with transition age 56 implied FIT testing at ages 50, 52, and 54, and colonoscopy at 56 and 66.

#### Risk score–based strategies

In risk score–based strategies, individuals underwent 1 or more QCancer risk assessments in addition to the screening tests. Each strategy was defined by a specific start age, stop age, interval for risk assessment, and a particular risk score percentile ranging from the top 10% to the top 90%. Upon each risk assessment, individuals were classified as high risk if their QCancer-predicted risk fell within the specified top risk percentile among all individuals assessed in that strategy. Given that QCancer risk increases strongly with age, individuals identified as high risk were predominantly older.^29^ We followed the exclusion criteria of the PRESENT trial by assuming that individuals did not report ulcerative colitis or previously detected polyps during their risk assessment, because these are exclusion criteria for routine CRC screening.^21^^,^^29^

For instance, in the strategy with risk assessments at ages 54, 64, and 74 and a 20% proportion of high-risk individuals, denoted as “20%_54:64:74”, all individuals started biennial FIT at age 50. QCancer risk was assessed at ages 54, 64, and 74, and individuals switched to colonoscopy once their risk score achieved the top 20% percentile of all individuals aged 54, 64, and 74. See Appendix S2 for further details. Note that age-based strategies are equivalent to risk score–based strategies with a single risk assessment and a 100% proportion of high-risk individuals.

#### FIT-based strategies

In FIT-based strategies, all individuals started in the low-risk group. Once they had an “elevated but negative” FIT result, they transitioned to colonoscopy at the next due screening, that is, 2 years after this FIT. This is consistent with biennial screening, including the Swiss policy of reimbursing a new screening test 2 years after a negative FIT. We evaluated 2 thresholds for defining elevated but negative FIT values: 5 μg/g (FIT5) and 10 μg/g (FIT10).

Additionally, we investigated FIT-based strategies with fixed transition ages. Participants that had not received an elevated but negative FIT result by this transition age were reclassified as high risk from that age onward. We evaluated all transition ages between 54 and 74 with 2-year increments in combination with the 5 and 10 μg/g thresholds.

For example, in the FIT-based strategy with FIT10 and transition age 66, individuals started FIT screening at age 50. If their FIT result was between 10 and 15 μg/g at, for instance, age 58 they were reclassified as high risk and they transitioned to colonoscopy by age 60. Individuals without a FIT result above 10 μg/g before age 66 transitioned to colonoscopy at age 66.

#### Reference strategies

As a comparison, we simulated a colonoscopy-only strategy where all participants underwent colonoscopy every 10 years (ages 50, 60, and 70), a FIT-only strategy with biennial FIT between ages 50 and 74, and a strategy in which half of the population underwent FIT and the other half colonoscopy from age 50 (denoted as 50/50). This strategy reflected a free choice program, like in the canton of Vaud, Switzerland.^12^ We assumed no transitioning between FIT and colonoscopy in these reference strategies.

### Model assumptions

In all strategies, participants with a positive FIT result (≥15 μg/g) were directly referred for a diagnostic colonoscopy. We assumed that once individuals had a colonoscopy, either due to a positive FIT or a high-risk classification, they would not switch back to FIT screening. If adenomas were found during colonoscopy, individuals entered colonoscopy surveillance according to Swiss guidelines.^30^ Surveillance was terminated at age 86. In the base case, we assumed full adherence to all screening recommendations, suggestions for FIT or colonoscopy, diagnostic colonoscopies, and surveillance. Simulated individuals yielded 1 QALY for every year they lived without CRC diagnosis. Reductions in QALYs (disutilities) assumed for screening tests and CRC treatment were in line with previous analyses (Tables S3.1 and S3.2).^10^ Assumed sensitivity and specificity of colonoscopy^10^ and FIT^31^ were obtained from previous studies, and colonoscopy complication rates were based on the results from the Vaud screening program^12^ (Table S3.3).

### Analysis

All strategies were evaluated by simulating a cohort of 5 million males and females for each underlying CRC risk group (see Appendix 3 in our previous publication^29^). For each strategy, we counted the outcomes of all simulated individuals alive and without detected CRC by age 50, independent of the switch age from FIT to colonoscopy. We evaluated QALYs gained and colonoscopy resources required for screening, surveillance, and clinical diagnoses compared to a scenario without screening. Outcomes were reported per 1000 50-year-olds without prior CRC diagnosis. Outcomes were not discounted in the base case, in line with previous analyses for the United States Preventive Service Task Force (USPSTF).^24^^,^^32^

Next, we identified *efficient* strategies within each of the 3 strategy types: age-based, risk score–based, and FIT-based. Efficient strategies are those yielding the highest benefits (QALYs) for a given colonoscopy requirement. The *efficiency frontier* is the line connecting efficient strategies when plotting QALYs and colonoscopy requirements for each strategy. For the efficiency frontier of risk score–based strategies, we also included age-based strategies because they can be considered specific risk score–based strategies. Finally, we identified an *overall efficiency frontier*—the line that connects the efficient strategies when considering all 3 strategy types simultaneously.

To account for stochastic variations in our results, we also considered *near-efficient* strategies—those that fell within 1.5 quality-adjusted months of the efficiency frontier—as efficient.^24^^,^^32^ Hereafter, the terms *efficient* and *efficiency frontier* include both efficient and near-efficient strategies.

### Sensitivity analyses

Consistent with the PRESENT trial results, sensitivity analyses first assumed 14% of the population to choose FIT and 14% colonoscopy, even if they were suggested otherwise, and 50% never participated in screening, independent of their CRC risk.^21^ The remaining 22% fully complied with their risk-based suggestion for FIT or colonoscopy. Second, we discounted QALYs gained by 3% annually starting at age 50. Third, we varied the screening stop age to mimic variation in opportunistic screening after the recommended stopping age in the target population.^33^ We evaluated 3 fixed stop ages (ages 70, 72, and 76), delayed screening cessation (one-third of individuals stops at ages 74, 76, and 78, respectively), and early and delayed cessation (one-fifth stops at ages 70, 72, 74, 76, and 78, respectively). The screening stop age was assumed independent from CRC risk. Finally, we modeled a screening start and stop age of 45 and 75, respectively, in line with USPSTF guidelines for colorectal cancer screening in the United States.^5^ These results were aggregated from age 45 instead of 50. For each sensitivity analysis we evaluated the same screening strategies as in the base case.

## Results

### Age-based strategies

**Figure 1:**
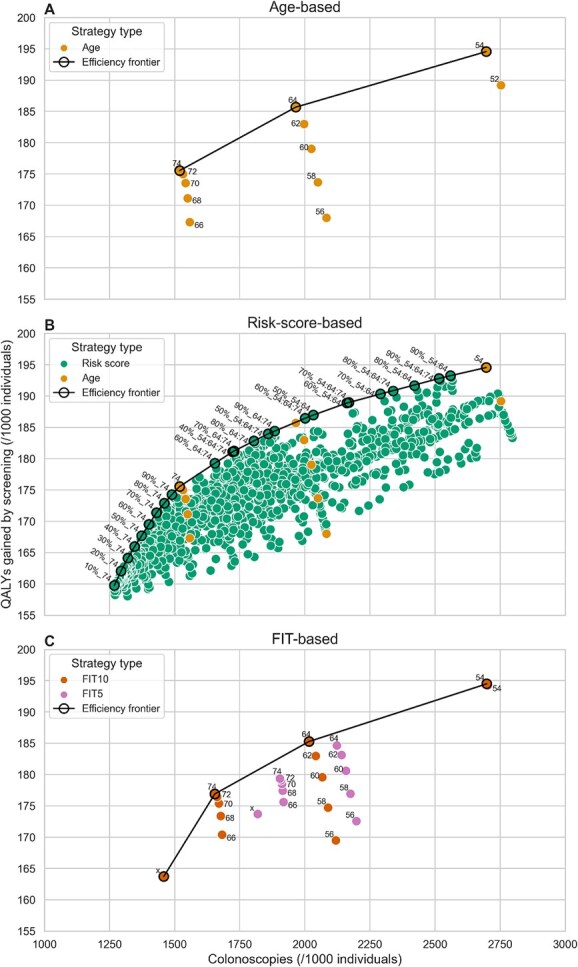
QALYs gained and colonoscopy demand by strategy type, compared to a no screening strategy. A strategy to the upper-left of another strategy is more efficient (more benefits, fewer colonoscopies). The efficiency frontiers, defined as the line connecting the efficient strategies, is represented by black lines. (A) Age-based strategies: individuals switch from FIT to colonoscopy at a specific age. The label number corresponds with the transition age of the strategy. (B) Risk score- and age-based strategies: only efficient strategies are labeled. The percentage corresponds with the fraction of individuals suggested colonoscopy, the numbers separated by colons reflect the risk assessment ages. For example, in strategy 40%_54:64, risk assessments are performed at ages 54 and 64, and the 40% of the 54- and 64-year-old population with highest risk scores switches to colonoscopy. (C) FIT-based strategies: individuals switch from FIT to colonoscopy if their most recent FIT result was between 5-15 μg/g (FIT5) or 10-15 μg/g (FIT10) in strategies labeled with “x”. In strategies labeled with an age, individuals transitioned from FIT to colonoscopy if they had not had such a FIT result by that age.

For age-based strategies, colonoscopy requirements are highly dependent on the number of lifetime colonoscopies (Figure 1A and Appendix S4). Transition ages 52-54 entailed 3 lifetime colonoscopies, thus requiring the most colonoscopies, whereas ages 66-74 required only 1 lifetime colonoscopy. Transition ages 54, 64, and 74 were efficient, yielding the most QALYs gained for colonoscopy demand, while ages 56 and 66 yielded the fewest QALYs gained for colonoscopy requirements.

### Risk score–based strategies

With 1268 to 1519 colonoscopies available per 1000 50-year-olds without CRC diagnosis, it was most efficient to suggest FIT until age 72, followed by a colonoscopy at age 74 for an increasing proportion of the population, with the remainder continuing FIT screening (Figure 1B). If available resources exceeded 1519, all participants could undergo colonoscopy by age 74 as part of the risk score–based strategy. With further increasing colonoscopy capacity, risk assessments were ideally performed at ages 64 and/or 54, with an increasing proportion of individuals classified as high risk by these ages. Ultimately, if the colonoscopy capacity exceeded 2698 colonoscopies, screening all participants with colonoscopy from age 54 yielded the most QALYs.

### FIT-based strategies

The FIT-based efficiency frontier only consisted of FIT10 strategies with a fixed transition age (Figure 1C). With increasing colonoscopy capacity, it would be most efficient to transition to colonoscopy at age 74, 64, or 54 if participants had not had a FIT result between 10 and 15 μg/g by then. The FIT10 strategy without transition age was not efficient.

### All strategies

**Figure 2:**
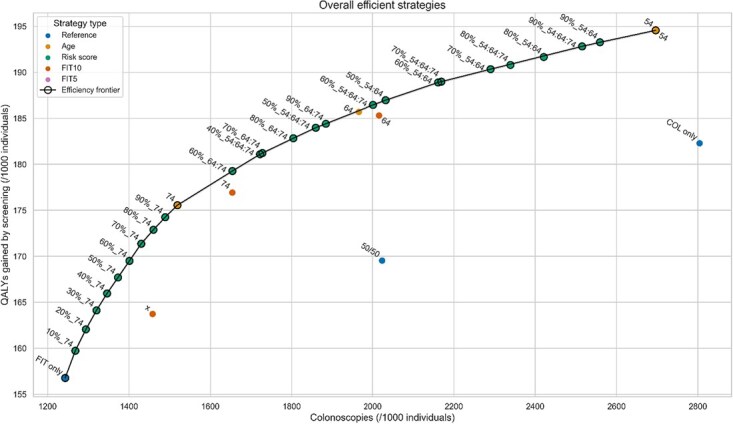
QALYs gained and colonoscopy demand of the efficient strategies within age-based, risk score–based and FIT-based strategies and the 3 reference strategies, compared to a no screening strategy. The black line marks the overall efficiency frontier (the set of efficient strategies when considering all three strategy types). For age-based strategies, the label number corresponds with the transition age of the strategy. For risk score–based strategies, the percentage corresponds with the fraction of individuals guided to colonoscopy, whereas the numbers separated by colons represent the risk assessment ages. For example, in strategy 40%_54:64, risk assessments are performed at ages 54 and 64, and the 40% of the 54- and 64-year-old population at highest risk is guided to undergo colonoscopy. For FIT-based strategies, the label number corresponds with the transition age of the strategy. Points labeled with “x” represent the FIT-based strategies without transition age.

When considering all strategies, the efficiency frontier consisted of age-based, risk score–based, and the FIT-only reference strategy (Figure 2, Table 2). The FIT-only strategy required the fewest colonoscopies and yielded fewest QALYs gained (1243 colonoscopies at 157 QALYs gained). As colonoscopy resources increased, a greater proportion of individuals should switch to colonoscopy by age 74 to achieve efficiency. With further increases in colonoscopy capacity, more individuals should switch at a younger age (age 64 or 54).

**Table 2:** Outcomes of the efficient strategies within each strategy type, as shown in Figure 2.

**Strategy type**	**Age-based switch criteria**	**Risk score– or** **FIT-based switch criteria**	**Number of colonoscopies (/1000 individuals)**	**QALYs gained** **(/1000 individuals)**	**Overall efficient strategy**
Reference	FIT only	n/a	1243	156.77	Yes
Risk score	74	10%	1268	159.73	Yes
Risk score	74	20%	1294	162.05	Yes
Risk score	74	30%	1320	164.12	Yes
Risk score	74	40%	1346	165.96	Yes
Risk score	74	50%	1373	167.69	No
Risk score	74	60%	1401	169.50	Yes
Risk score	74	70%	1430	171.36	Yes
FIT10	x	10 μg/g	1458	163.72	No
Risk score	74	80%	1460	172.88	Yes
Risk score	74	90%	1489	174.25	Yes
Age	74	n/a	1519	175.55	Yes
FIT10	74	10 μg/g	1654	176.92	No
Risk score	64, 74	60%	1654	179.26	Yes
Risk score	54, 64, 74	40%	1722	181.09	Yes
Risk score	64, 74	70%	1728	181.22	Yes
Risk score	64, 74	80%	1804	182.83	Yes
Risk score	54, 64, 74	50%	1859	183.97	Yes
Risk score	64, 74	90%	1884	184.40	No
Age	64	n/a	1965	185.69	No
Risk score	54, 64, 74	60%	2001	186.45	Yes
FIT10	64	10 μg/g	2016	185.30	No
Reference	50/50	n/a	2023	169.52	No
Risk score	54, 64	50%	2032	186.96	Yes
Risk score	54, 64	60%	2161	188.89	Yes
Risk score	54, 64, 74	70%	2169	188.98	Yes
Risk score	54, 64	70%	2290	190.33	Yes
Risk score	54, 64, 74	80%	2339	190.80	No
Risk score	54, 64	80%	2421	191.66	No
Risk score	54, 64, 74	90%	2515	192.82	Yes
Risk score	54, 64	90%	2559	193.26	Yes
Age	54	n/a	2696	194.56	Yes
FIT10	54	10 μg/g	2698	194.50	No
Reference	COL only	n/a	2804	182.28	No

The last column indicates which strategies are overall efficient, that is, efficient when considering all 3 types of strategies. n/a: not applicable.

The colonoscopy-only reference strategy was not efficient. By adopting the age-based strategy with transition age 54, QALYs gained would increase by 6.7% (194.6 vs 182.3) while colonoscopy requirements decreased by 3.9% (2696 vs 2804). Alternatively, a risk score–based strategy could decrease colonoscopy demand by 35.7% (1804 vs 2804) while maintaining equal QALYs gained. The free choice strategy (50/50) was also not efficient. By recommending colonoscopy to the top 60% of risk scores among all 54-, 64-, and 74-year-old participants, QALYs gained from screening would increase by 10.0% (from 169.5 to 186.5 QALYs) with similar colonoscopy capacity (2023 vs 2001 colonoscopies). Alternatively, colonoscopy demand could be reduced by 30.7% (1401 vs 2023) without losing QALYs gained.

### Sensitivity analyses

Results of sensitivity analyses are provided in Appendix S5. Imperfect adherence to screening reduced colonoscopy demand and QALYs gained from all strategies with minimal impact on the efficiency frontier (Figure S5.1). Strategies 10%_70:72:74 and 10%_72:74 became efficient under these conditions. However, most individuals in these strategies would still transition to colonoscopy at age 74, since only a small proportion of 70- and 72-year-olds would be in the top 10% risk percentile, and only 44% of those would switch to colonoscopy. Annual discounting reduced QALYs gained for all strategies and had a similar impact on the overall efficiency frontier as the imperfect adherence assumptions (Figure S5.2 and Table S5.1).

With varying screening stop ages, FIT-based strategies remained off the efficiency frontiers. For fixed stop ages, the optimal ages to switch (age-based strategies) or perform a risk assessment (risk score–based strategies) were at screening cessation, or 10 or 20 years prior, like in the base case (Figures S5.3-S5.5). Notably, the colonoscopy-only strategy was efficient if screening stopped at age 50. With delayed screening cessation, the efficient ages for switching or risk assessment remained at 54, 64, and 74 (Figure S5.6).

With both delayed and early screening cessation, age-based and risk score–based strategies remained most efficient (Figure S5.7 and Table S5.2). The efficient ages for switching or risk assessments were at ages 50, 60, and 70. For lower capacities, however, risk assessments would ideally occur at ages 70, 72, and/or 74. This is because by age 72 and 74, more individuals have died or stopped screening than at age 70, making it feasible to screen a subset of 74-year-olds with limited colonoscopy capacity. With screening initiation and cessation according to the USPSTF guidelines, it was efficient to switch at ages 55, 65, or 75, or perform colonoscopy only (Figure S5.8 and Table S5.3).

## Discussion

This study predicts that CRC screening strategies where all individuals initially use biennial FIT and some later switch to decennial colonoscopy generally outperform colonoscopy-only screening, and a strategy where an equal proportion of individuals choose FIT or colonoscopy at age 50 without switching. Specifically, if screening stops at age 74, it is most efficient to transition from FIT to colonoscopy at ages 54, 64, or 74, based on age or risk scores. This may increase QALYs by up to 10.0% or reduce the number of colonoscopies by up to 30.7%. Transitioning at ages 56 or 66 was least efficient. For other screening stop ages, transitions are also ideally scheduled at the screening stop age or 10-yearly increments before this age. The FIT-based suggestions were not part of the overall efficiency frontier.

Our results highlight the importance of the age at which individuals undergo their final CRC screening test, even in risk-based strategies. The colonoscopy-only and 50/50 reference strategies were not efficient because the individuals undergoing colonoscopy screening stop screening at age 70, 4 years before the upper age limit of 74. This premature cessation is caused by the 10-year screening interval for colonoscopy: a repeat colonoscopy after age 70 would occur at age 80, which exceeds the upper age limit for screening. Hence, the last screening test is performed at age 70, and this gives room for additional FITs. The FIT-only strategy and hybrid strategies that begin with FIT and transition to colonoscopy at ages 54, 64, or 74 ensure that the final test is performed at age 74, and therefore achieve efficiency. In contrast, transitions at ages 56 or 66 are inefficient: the final colonoscopy would occur at age 66 because the colonoscopy 10 years later would be after the stopping age, effectively stopping screening 8 years early. In general, given the 10-year interval between colonoscopies, the most efficient strategies are those that schedule transitions 0, 10, or 20 years before screening cessation. This way, screening covers the full eligible screening age range. Consequently, the colonoscopy-only strategy appears only on the efficiency frontier in sensitivity analyses where the start and stop age of screening aligned with the 10-year colonoscopy interval (45-75 and 50-70). Our result that the 50/50 reference strategy does not achieve efficiency in sensitivity analyses underscores that older individuals should be prioritized over younger individuals for colonoscopy screening in health care settings with limited colonoscopy capacity.

Our results also clearly suggest that risk score–based approaches add limited value beyond age alone in guiding the timing of transitions to colonoscopy. Across all analyses, the efficiency frontier essentially followed a straight line between age-based strategies with transitions 0, 10, or 20 years prior to screening cessation. Even in sensitivity analyses with nonuniform screening stop ages, age-based approaches performed as well as—or better than—risk score–based and FIT-based approaches.

We are unaware of other studies that modeled the long-term impact of risk-based recommendations for colonoscopy or FIT. However, previous studies have predicted benefits from strategies where individuals switch from FIT to colonoscopy.^14^^,^^15^^,^^24^ One of these contrasted our findings and predicted that initiating FIT at age 50 followed by colonoscopies at 55 and 65 would be more effective than FIT followed by colonoscopies at 60 and 70.^15^ This likely stems from their assumption that adenomas progress more slowly to CRC, resulting in a longer protective effect of colonoscopy. Other modeling studies also predicted that a start age, end age, and/or interval based on risk scores hardly improves effectiveness compared to age-based screening in realistic strategies.^34^^-^^36^ Specifically, our previous study predicted that screening initiation based on QCancer did not outperform initiation based on age and sex.^29^

However, the unfavorable results of our FIT-based approaches contrast markedly with the recent trend toward risk-based CRC screening using negative prior FIT results.^37^^-^^39^ This discrepancy has 2 explanations. First, elevated FIT values indicate a short-term potential risk of CRC or advanced neoplasia and may need timely interventions such as advancing the invitations for the next screening test. In our FIT-based strategies, individuals are not invited earlier: their colonoscopy remains scheduled after 2 years. During this period, the disease may further progress. Some individuals may develop an interval cancer in these 2 years and do not benefit from the FIT-based strategy. Others may have a positive FIT after 2 years resulting in a colonoscopy after 2 years, even in strategies without FIT-based switch. On the other hand, our FIT-based approaches result in transitions to colonoscopy at suboptimal ages, leading to earlier screening cessation. For example, individuals with elevated but negative FIT results at age 64 receive their final colonoscopy at 66 and are disadvantaged by the early screening cessation. Overall, the benefits experienced by the relatively small group who gain from their FIT-based transition do not outweigh the disadvantages of those with early screening cessation. This suggests that risk-based CRC screening based on prior FIT results may be better suited to inform a change in FIT interval or positivity cut-off for subsequent screenings, rather than to guide transitions to colonoscopy for which a long screening interval is recommended.

A strength of this study is that we evaluated risk prediction approaches with high potential: using one of the best available CRC risk prediction tools^20^ or fecal hemoglobin concentrations, which is a promising biomarker. We used realistic screening scenarios, adhering to Swiss health insurance reimbursement policies. Moreover, we used adherence data from our pilot trial in a sensitivity analysis.^21^ We conducted sensitivity analyses with fixed and flexible screening stop ages, making our results generalizable to opportunistic (like the United States) and programmatic screening settings (like The Netherlands). Finally, due to its prospective design, our modeling analysis is not susceptible to immortal time bias: outcomes are aggregated from age 50 until death, regardless of the switch age from FIT to colonoscopy.

Our study also has limitations. First, the assumed disutilities, complication rates, test characteristics, and screening adherence rates did not depend on age and time although they may be age- and time-dependent in reality. For example, older individuals and those with comorbidities experience greater physical burden and complication risk for colonoscopy, preventing them from undergoing colonoscopy.^40^ Moreover, the sensitivity and specificity of FIT worsen with increasing age.^41^ With these factors accounted for, strategies using elevated but negative FIT values or risk prediction tools could be more efficient. Second, we assumed perfect adherence in our base case analysis. Especially in opportunistic screening settings, adherence to the recommended screening intervals is imperfect, and colonoscopies are often done after the recommended screening stop age. However, varying screening stop ages did not change our results. Additionally, using imperfect adherence in policy optimization studies leads to overscreening in guideline-concordant individuals.^42^ Third, we did not incorporate health care costs because they are difficult to obtain in Switzerland. Our results cannot be used for a formal decision analysis as cost-effective strategies might be different from our efficient strategies. Fourth, the large number of simulation runs did not permit deeper uncertainty analyses. Stochasticity in model results was handled by the concept of near-efficiency, like in USPSTF modeling analyses.^24^^,^^32^ Finally, available capacity for screening colonoscopies does not imply that the full capacity should be used in practice. Our model only accounted for colonoscopies needed for screening, surveillance, and clinical diagnoses, while capacity must support nonscreening exams.

We are cautious to generalize our outcomes to other countries with different demographics or risk factor distributions. However, the fact that the QCancer tool relies primarily on age and sex, and FIT performance characteristics are likely comparable across countries. The relative effectiveness of the age-based, risk score–based, and FIT-based strategies therefore presumably generalizes to other settings such as the United States, and age-based strategies are also unlikely to be outperformed there. Our results also extend to settings where other noninvasive screening tests such as multitarget stool DNA are used instead of FIT. Given that these tests have comparable effectiveness and invasiveness to annual FIT,^43^ such settings follow the same principle as in our analyses: initiating screening with a less sensitive test at shorter intervals and transitioning to decennial colonoscopy.

As a consequence, our results have important implications for risk-based screening practice. First, they showed that in settings with a free choice between FIT and colonoscopy, like in Switzerland and the United States, screening efficiency can simply be improved with age-based recommendations for FIT or colonoscopies. Individuals should start with FIT screening and switch to colonoscopy such that the last colonoscopy occurs close to screening cessation. Shared decision-making should apply here, allowing participants to deviate from the suggestions in consultation with their primary care provider. Second, programs considering a colonoscopy-only approach might improve screening efficiency by initially inviting individuals for FIT and transitioning to colonoscopy at a later, suitable age. Third, our study illustrates that besides research on biomarkers and risk prediction algorithms, policymakers and scientists should also investigate the optimal implementation of such risk factors and algorithms. Although our study used one of the best performing CRC risk prediction methods, age alone remained the most relevant criterion because of the way these tools were used in our simulated screening strategies. Therefore, results from ongoing randomized trials and modeling studies should help to guide and optimize the implementation of risk-based CRC screening programs.^38^^,^^39^^,^^44^^,^^45^

To conclude, screening guidelines offering a choice between 10-yearly colonoscopy and biennial FIT, or offering colonoscopy only, can improve efficiency by simply guiding participants to colonoscopy at ages 0, 10, or 20 years before screening ends to ensure that their final colonoscopy aligns with screening cessation. Complex approaches based on QCancer risk scores or prior FIT values do not outperform age-based approaches as the age of the final screening test strongly affects screening efficiency.

## Supplementary Material

Web_Material_kwaf214

## Data Availability

Data can be obtained from the corresponding author upon reasonable request.
