# Supplementary material for: Risk-based guidance for choosing fecal immunochemical test or colonoscopy in colorectal cancer screening: a modeling study
Source: Am J Epidemiol. 2025 Sep 30;195(3):832–40. doi: 10.1093/aje/kwaf214 (PMC13017602; doi:10.1093/aje/kwaf214)
Supplement: Web_Material_kwaf214 [file web_material_kwaf214.zip › 20250922_FitVsCol_Appendix.docx]

APPENDICES

to

**Risk-based guidance for choosing FIT or colonoscopy in colorectal cancer screening: a modelling study**

Authors:

Luuk A. van Duuren, Jean-Luc Bulliard, Matthias Harlass,

Ekaterina Plys, Douglas A. Corley, Florian Froehlich,

Kevin Selby, Iris Lansdorp-Vogelaar

Included materials

[Appendix S1 – Adapting MISCAN-Colon to Switzerland 2](#_Toc209436867)

[Appendix S2 – Overview of risk-score-based strategies 6](#_Toc209436868)

[Appendix S3 – Assumed disutilities and test characteristics 8](#_Toc209436869)

[Appendix S4 – Complete results of base case analysis 10](#_Toc209436870)

[Appendix S5 – Results of sensitivity analyses 10](#_Toc209436871)

[References 21](#_Toc209436872)

# Appendix S1 – Adapting MISCAN-Colon to Switzerland

MISCAN-Colon is a stochastic microsimulation model. The model has been described extensively in previous publications.^1,2^ To develop a reliable version of MISCAN-Colon for our study, we adapted and recalibrated the model to Switzerland, similar to the approach by Gini et al.^3^

## S1.1 – Calibration procedure

We started using the previously calibrated model for The Netherlands and adjusted specific demographics and CRC epidemiological assumptions to create sex-specific models for Switzerland:

- We used the all-cause mortality tables from 2019 (before COVID) from the Federal Statistical Office of Switzerland;
- We incorporated the CRC subsite distribution using data from 1985-1989, before (opportunistic) CRC screening occurred, obtained from the National Agency for Cancer Registration (NACR). It included data from six registries covering nine out of the 26 Swiss cantons: both Appenzell (AR, AI), Basel City and Land (BS, BL), Geneva (GE), Neuchâtel (NE), Sankt-Gallen (SG), Vaud (VD), and Zurich (ZH).
- We adjusted the input parameters for CRC survival by comparing the subsite (colon and rectum) and stage-specific 5-year relative survival observed in The Netherlands and Switzerland in the period 2014-2018. The ratio between the two survival rates was subsequently used as a multiplicative factor to adjust the MISCAN-Colon age-, stage- and subsite-specific CRC relative survival model parameters from the Dutch MISCAN-Colon model. The Swiss survival rates were obtained from NACR and included all Swiss cantons except Aargau (AG), Freiburg (FR), Schaffhausen (SH) and Schwyz (SZ).

Next, we calibrated two sets of model parameters. First, we assumed a similar biology (cancer pathway) for CRC development in The Netherlands and Switzerland. This implied that the difference in CRC incidence between the two countries is only explained by a difference in risk of adenoma onset. We calibrated the parameters for age-specific risk of adenoma onset such that the model aligned with the Swiss CRC incidence. Second, we assumed that the difference in CRC stage distribution between The Netherlands and Switzerland was caused by differences in access to care, in absence of screening, between the two countries. We therefore recalibrated the probabilities of CRC diagnosis in each stage to the Swiss CRC stage distribution. A genetic algorithm was used for calibration.^4^

- We used the Swiss CRC incidence from the period 1985-1989 as calibration targets, obtained from NACR for the nine Swiss cantons mentioned earlier.
- We also used the Swiss CRC stage distribution by subsite (left colon, right colon and rectum) from the period 1985-1989 as calibration target. We only used data from the registry of the canton of Geneva, because it was the only one with sufficiently complete data.

Finally, we validated the model by comparing the model-predicted CRC mortality with the observed CRC mortality in Switzerland.

- We used the total Swiss CRC mortality from the periods 1985-1989, 1995-1999 and 2010-2014 as validation targets. Data were obtained from NACR and included the whole of Switzerland.
- We adjusted the model parameters for CRC survival to the time periods 1985-1989 and 1995-1999. As described before, we compared the subsite-specific CRC survival (colon and rectum) from Switzerland from these periods^5,6^ with the Swiss CRC survival from the period 2010-2014.

## S1.2 – Calibration results

Figures S1.1 and S1.2 show the calibration results for the age-specific CRC incidence of the models for females and males, respectively. The black dots with confidence intervals are the observed Swiss data, the red line represents the model output. Although slightly underestimating the incidence for the 80-84 and 85+ age groups, the model-predicted incidence fits the pattern of the observed incidence well.


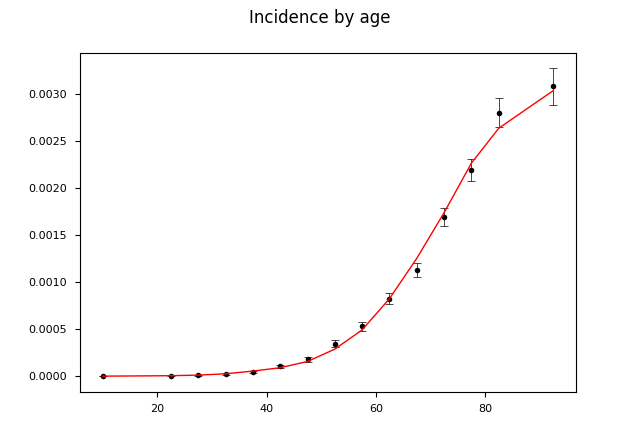


*Figure S1.1 – The model-predicted and observed age-specific CRC incidence rates per 100,000 females in Switzerland.*


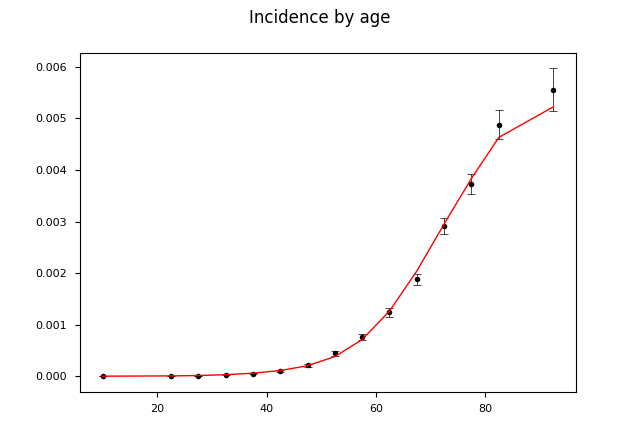


*Figure S1.2 – The model-predicted and observed age-specific CRC incidence rates per 100,000 males in Switzerland.*

## S1.3 – Validation results

Figure S1.3 shows the overall mortality rate as predicted by MISCAN and observed in the Swiss population. Remarkably, the model highly underestimates the CRC mortality in 1985-1989. However, until 1995, CRC mortality in Switzerland included all individuals that died **with** CRC whereas we modelled individuals that died **of** CRC.^7^ The Swiss coding protocol was changed in 1995, and we observe that our model is able to replicate mortality in the period 1995-1999, slightly overestimating mortality rates between ages 70-84. Compared to 1995-1999, the model overestimates CRC mortality more in 2010-2014. This is possibly due to (opportunistic) CRC screening introduction in Switzerland by that time, and we did not incorporate screening in MISCAN for this validation procedure.


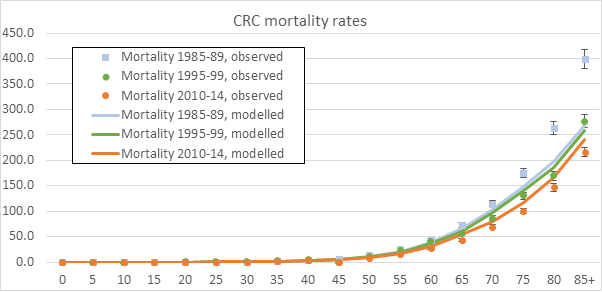


*Figure S1.3 – Model-predicted and observed age-specific CRC mortality rates per 100,000 persons in Switzerland for the 5-year periods 1985-1989, 1995-1999 and 2010-2014.*

Appendix S2 – Overview of risk-score-based strategies

Risk-score-based strategies are characterized by 1) a set of ages and 2) a percentile. First, the set of ages specifies when individuals complete risk assessments and evaluate their QCancer-predicted risk. Each set of ages yields a unique distribution of QCancer-predicted risk in the tested population. We previously derived this distribution using data from the Swiss Health Survey, an individual-level health survey that is representative of the Swiss population.^8,9^ We estimated the distribution of QCancer risk by age and sex, accounting for changes in risk factor prevalence between age and sex (e.g. smoking behavior, BMI distribution, etc.). However, to increase the sample size for this distribution, we assumed that the prevalence of QCancer risk factors (apart from age and sex) did not change within 5-year age groups. For example, the distribution of BMI, alcohol and smoking behavior was assumed not to change between ages 50 to 54, nor 55 to 59, etc. These distributions only changed at the boundaries between 5-year age groups, for example from age 54 to 55, or 59 to 60. Therefore, the changes in QCancer distributions between, for instance, ages 50 and 54 merely reflects aging, whereas changes between ages 54 and 55 also reflect changes over time in smoking behavior, BMI and other risk factors.

Then, to derive the distribution of QCancer risk in the tested population for a specific risk-score-based strategy, we took the following steps:

1. For each risk assessment age in the “set of ages” of the risk-score-based strategy, we considered all respondents to the SHS that were in the same 5-year age group. For each individual, we adjusted their age to the risk assessment age and calculated their QCancer risk. This resulted in a distribution of QCancer for the specific risk assessment age.
2. By combining the distributions obtained in step 1, we obtained the distribution of QCancer risk in the tested population for this risk-score-based strategy.

Next, using the obtained distribution of QCancer risk, we determined the threshold QCancer risk value to differentiate between high- and low-risk individuals based on the strategy’s percentile. The threshold was chosen such that the proportion of individuals above the threshold was equal to the percentile.

Consider, for example, the strategy “30%_54:62:70”. In this strategy, individuals have a risk assessment at ages 54, 62 and 70. Therefore, we considered all individuals in the Swiss Health Survey at ages 50-54, 60-64 and 70-74, adjusted their ages to 54, 62 and 70, respectively, and calculated their QCancer-predicted risk (i.e. we assumed that the prevalence of risk factors did not change within 5-year age groups).^10,11^ This resulted in an estimate of the distribution of QCancer risk for all Swiss individuals aged 54, 62 and 70 years old (Figure S2.1).

Next, we chose the threshold between high- and low-risk individuals such that 30% of the population aged 54, 62 and 70 had risk scores that exceed this value. Note that the majority of the high-risk population will be 70, some will be 62 and only a few will be 54 because QCancer highly depends on age (Figure S2.1). Consequently, of all individuals that are suggested to switch to colonoscopy at some point in their lives, most receive this suggestion by age 70, some by age 62 and only a few by age 54.

We assumed that once an individual’s QCancer risk exceeded the threshold, it would never get below the threshold again. Our previous publication gives further details on how the SHS data were used, and how QCancer risk distributions were combined with results from MISCAN simulations.^9^

We evaluated 1575 unique risk-based strategies: all unique and valid combinations of the properties in Table S2.1.

Qcancer-predicted risk

54

62

70

Density

Low-risk

(70%)

High-risk

(30%)

*Figure S2.1 – Illustration of the risk-score-based strategy 30%_54:62:70. The purple, red and orange distributions illustrate the distribution of QCancer-predicted risk in the Swiss population for the 54-, 62- and 70-year-olds, respectively. The vertical dashed line indicates the threshold between high- and low-risk scores, where 30% of this distribution has a high-risk score and 70% a low-risk score. In this illustration, most individuals will transition to colonoscopy by age 70, some by age 62 and only a few by age 54.*

*Table S2.1 – Overview of the risk-score-based screening strategies*

| **Start age of risk assessments** | **Stop age of risk assessments*** | **Interval between risk assessments (years)** | **High-risk percentile** | **Number of unique^#^, valid* strategies** |
| --- | --- | --- | --- | --- |
| 50, 52, 54, 56, 58, 60, 62, 64, 66, 68, 70, 72, 74 | 50, 52, 54, 56, 58, 60, 62, 64, 66, 68, 70, 72, 74 | 2, 4, 6, 8, 10 | 10%, 20%, 30%, 40%, 50%, 60%, 70%, 80%, 90% | 1575 |

**The stop age must be greater or equal to the start age for the strategy to be valid.*
*^#^Some combinations of start age, stop age and interval lead to non-unique strategies. For example, start age 50, stop age 52 and intervals 4, 6, 8 and 10 all schedule a risk assessment at age 50 only. Only one of these non-unique combinations was considered.*

# Appendix S3 – Assumed disutilities and test characteristics

*Table S3.1 – Assumed disutilities (reductions in QALYs) for CRC treatment.^12^*

|  | | **Phase of cancer care*** | | | |
| --- | --- | --- | --- | --- | --- |
|  |  | **Initial phase** | **Continuing phase** | **Terminal phase, death CRC** | **Terminal phase, death other causes** |
| **CRC stage** | **Stage I** | 0.12 | 0.05 | 0.70 | 0.05 |
|  | **Stage II** | 0.18 | 0.05 | 0.70 | 0.05 |
|  | **Stage III** | 0.24 | 0.24 | 0.70 | 0.24 |
|  | **Stage IV** | 0.70 | 0.70 | 0.70 | 0.70 |

**The terminal phase takes precedence over the initial and continuing phase. The terminal phase reflects the last 12 months of life and different disutilities are assumed for CRC and other-cause mortality. The initial phase reflects the at most 12 months following diagnosis for persons who survive for more than 12 months (if survival <12 months, the person only experience the terminal phase). The continuing phase is the time between the initial phase and the terminal phase for persons who survive more than 24 months.*

*Table S3.2 – Assumed disutilities (reductions in QALYs) for CRC screening tests.^12^*

| **Negative FIT** | **Positive FIT** | **Colonoscopy without polypectomy** | **Colonoscopy with polypectomy** |
| --- | --- | --- | --- |
| 0.00063 | 0.001330 | 0.000496 | 0.001401 |

*Table S3.3 – Assumed test characteristics for FIT and colonoscopy*

|  | **Colonoscopy** | **FIT - 15 µg/g ^a^** | | **FIT - 10 µg/g ^a^** | | **FIT - 5 µg/g ^a^** | |
| --- | --- | --- | --- | --- | --- | --- | --- |
|  |  | Males | Females | Males | Females | Males | Females |
| Specificity | 86% ^b^ | 92.11% | 93.35% | 89.33% | 90.87% | 83.00% | 85.06% |
| Sensitivity ^c^ Adenoma 1-5 mm ^d^ Adenoma 6-9 mm Adenoma 10+ mm Cancer (long) ^e^ Cancer (short) ^e^ | 75%  85% 95% 95%  95% | 0.00%  0.38% 23.2% 68.5% 91.3% | 0.00%  1.09% 22.4% 60.9%  88.2% | 0.00%  0.19%  25.3%  74.2%  93.3% | 0.00%  0.75%  24.5%  65.3%  90.0% | 0.00%  0.04%  29.9%  80.7%  95.2% | 0.00%  0.34%  29.1%  73.0%  92.9% |
| Caecal intubation rate | 94% | Not Applicable | | | | | |
| Probability of fatal complication after colonoscopy with polypectomy | 1.91 per  10 million ^f^ |  |  |  |  |  |  |
| Probability of hospitalization after colonoscopy with polypectomy | 0.07% ^g^ |  |  |  |  |  |  |

1. Assumed values for FIT sensitivity and specificity were based on a previously published meta-analysis.^13^
2. The lack of specificity for colonoscopy reflects the detection of non-adenomatous lesions which are removed. This induces polypectomy and/or biopsy which might lead to complications after colonoscopy. The specificity of FIT represents the fraction of individuals that are referred for follow-up colonoscopy, independent of whether they have a lesion.
3. The values are per-lesion sensitivities. The sensitivity of colonoscopy for the detection of adenomas and CRC within the reach of the endoscope was obtained from a systematic review on miss rates observed in tandem colonoscopy studies.^14^
4. MISCAN-Colon assumes that small adenomas cannot cause a positive stool test but can be accidentally found due to the lack of specificity by the test. So, despite 0% sensitivity for small adenomas, 6-17% of small adenomas test positive for lack of specificity, depending on sex and positivity cutoff.
5. MISCAN-Colon assumes a higher sensitivity for lesions “short” before clinical diagnosis compared to “long” before diagnosis. The higher sensitivity applies to lesions that will show clinical symptoms in their current TNM stage. The lower applies to lesions that will first progress to the next TNM stage before showing clinical symptoms.
6. Risk of dying from a colonoscopy with polypectomy.^2^
7. Based on the most recent evaluation of the CRC screening program in the canton of Vaud, Switzerland.^15^

# Appendix S4 – Complete results of base case analysis

The attached Excel sheet contains the outcomes (QALYs gained and colonoscopies required) of all simulated screening strategies in the base case analysis.

# Appendix S5 – Results of sensitivity analyses


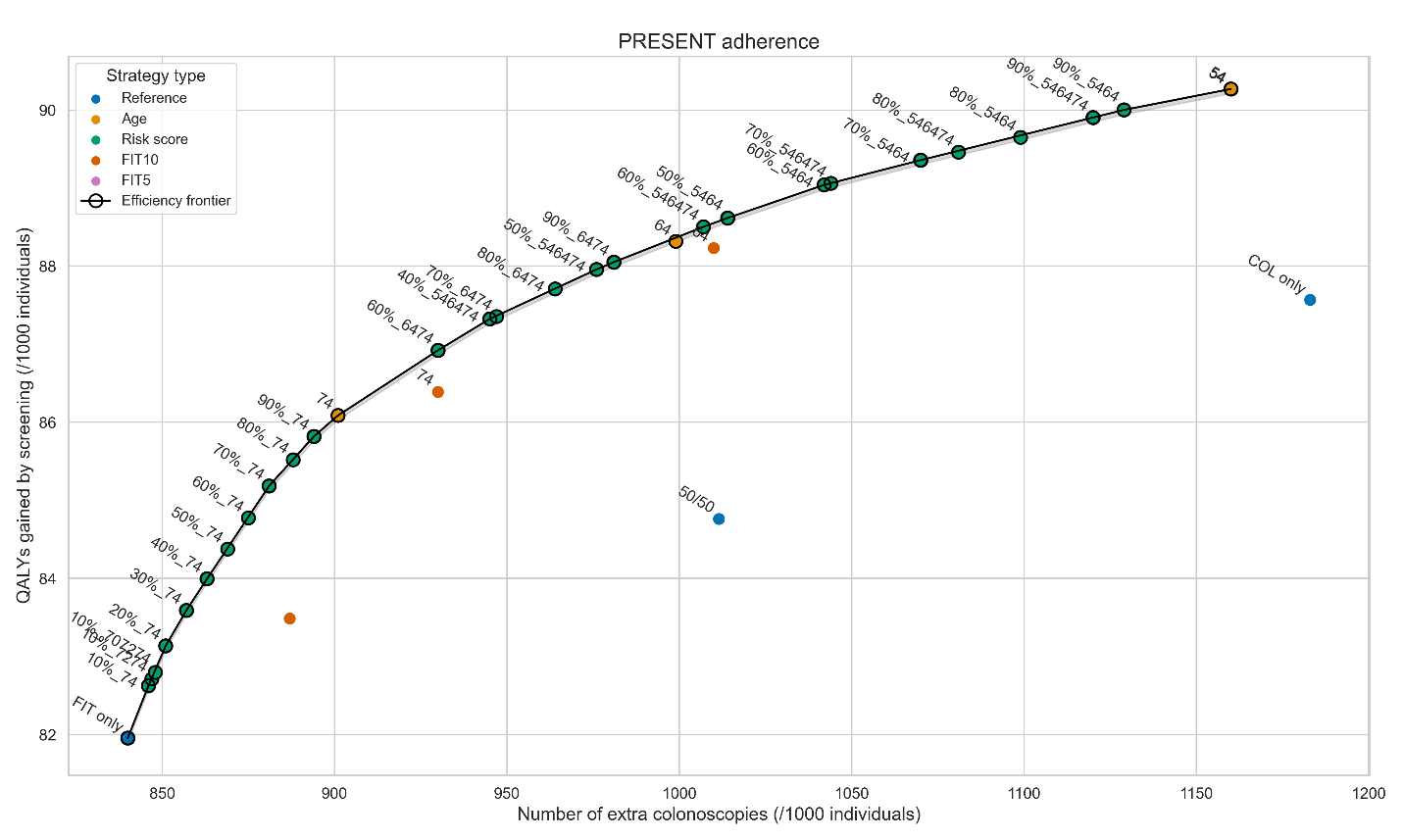
*Figure S5.1 – Overall efficient strategies in the sensitivity analysis assuming screening adherence according to the PRESENT trial.*


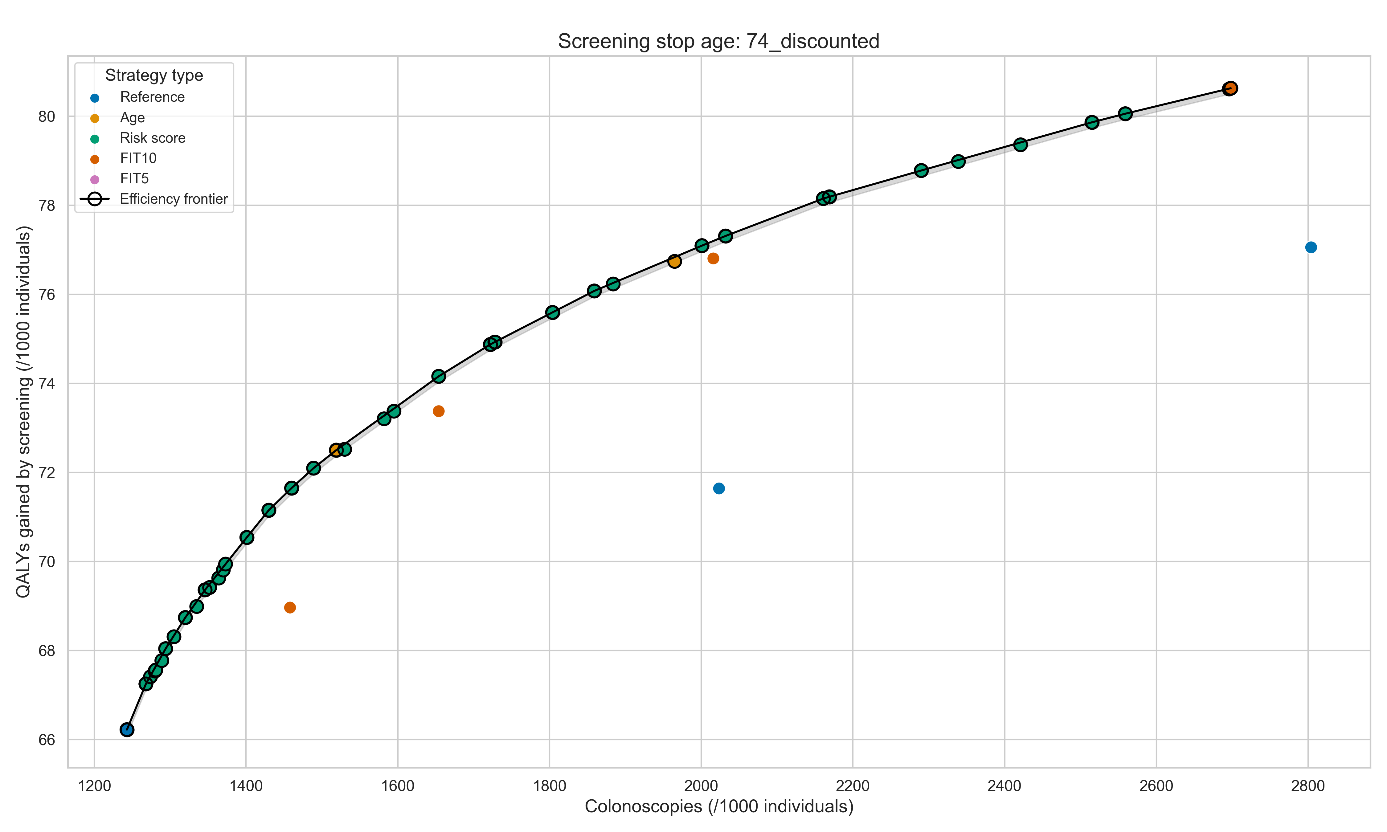
*Figure S5.2 - Overall efficient strategies in the sensitivity analysis in which QALYs gained are discounted. Labels are omitted due to a lack of space. Outcomes per strategy are shown in Table S5.1.*


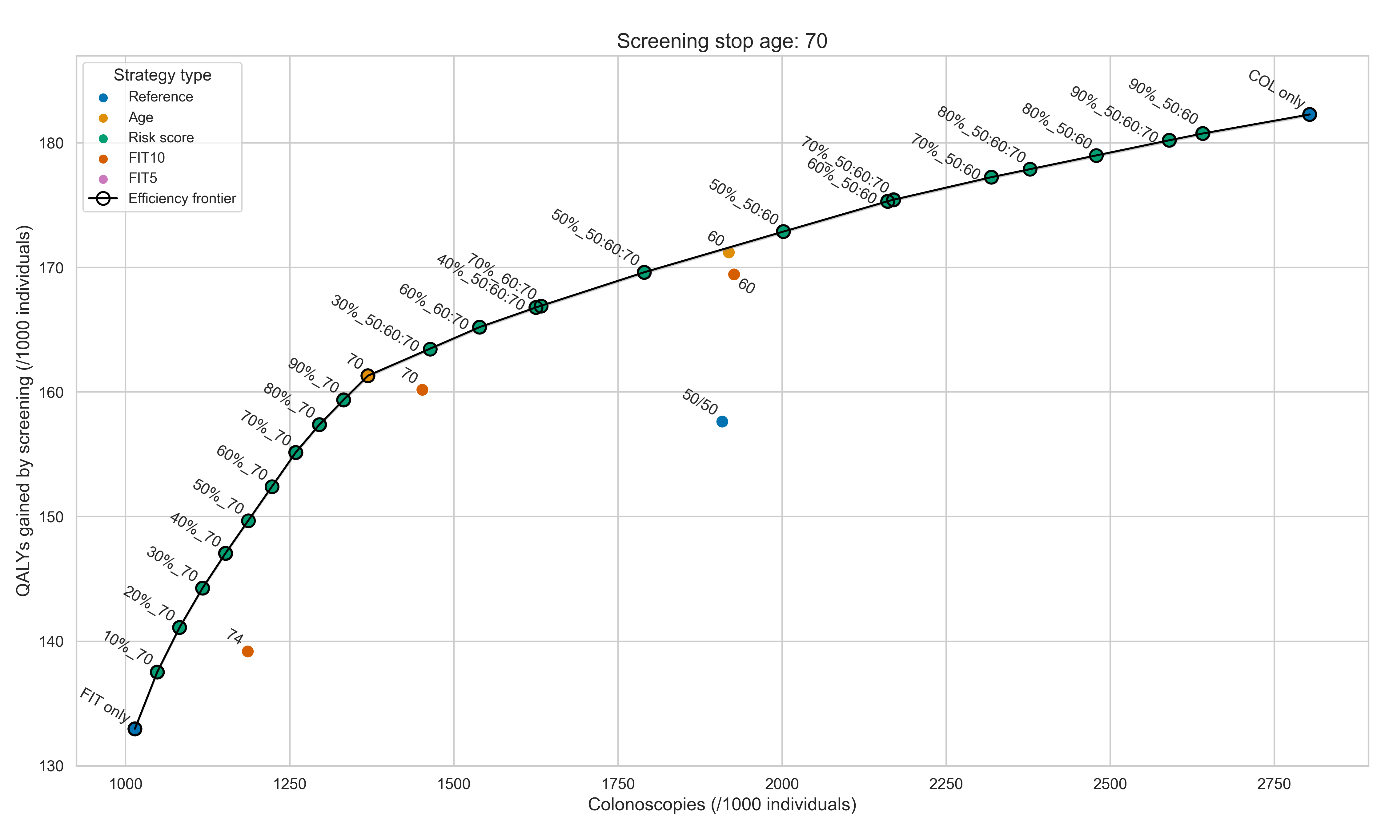
*Figure S5.3 – Overall efficient strategies in the sensitivity analysis with a screening stop age of 70.*


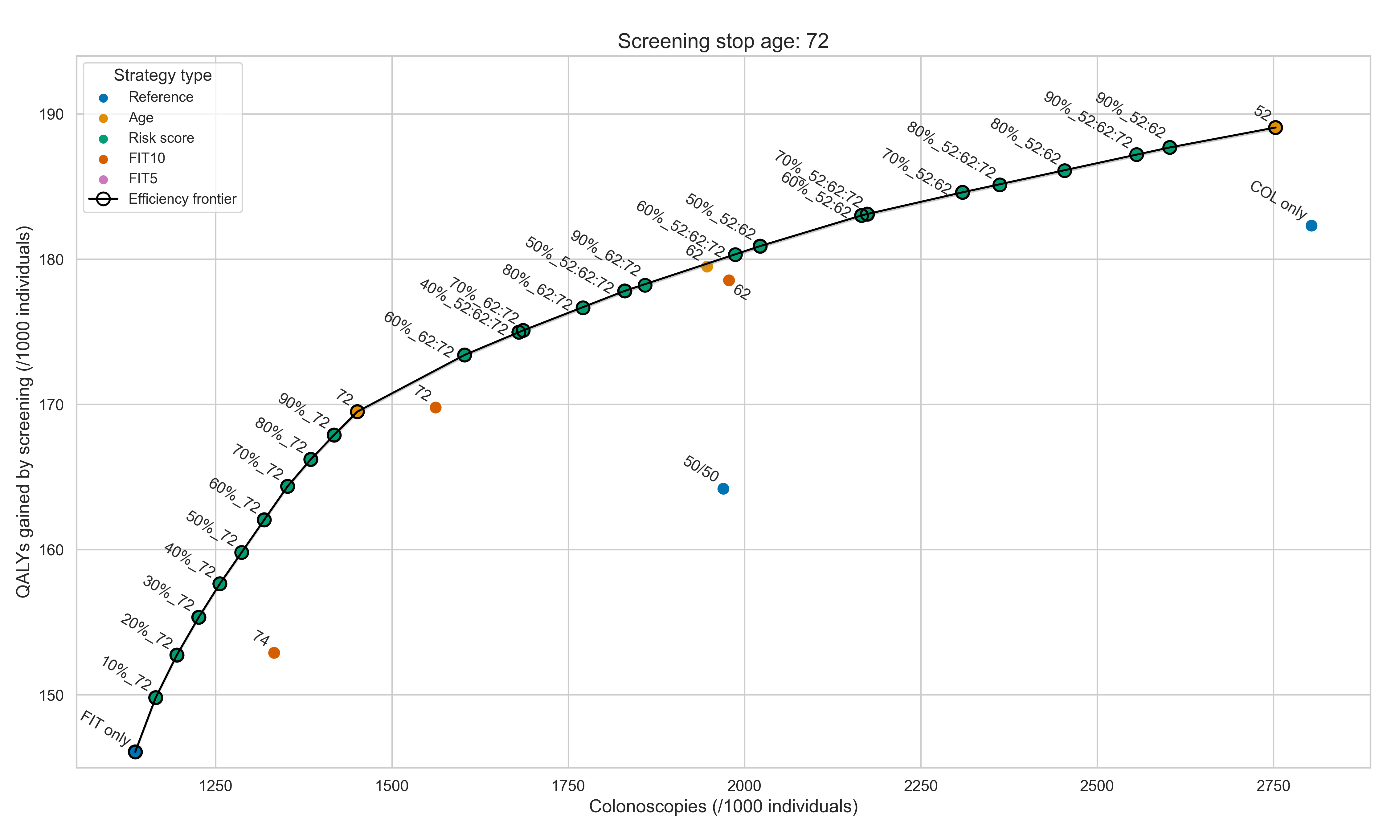
*Figure S5.4 – Overall efficient strategies in the sensitivity analysis with a screening stop age of 72.*


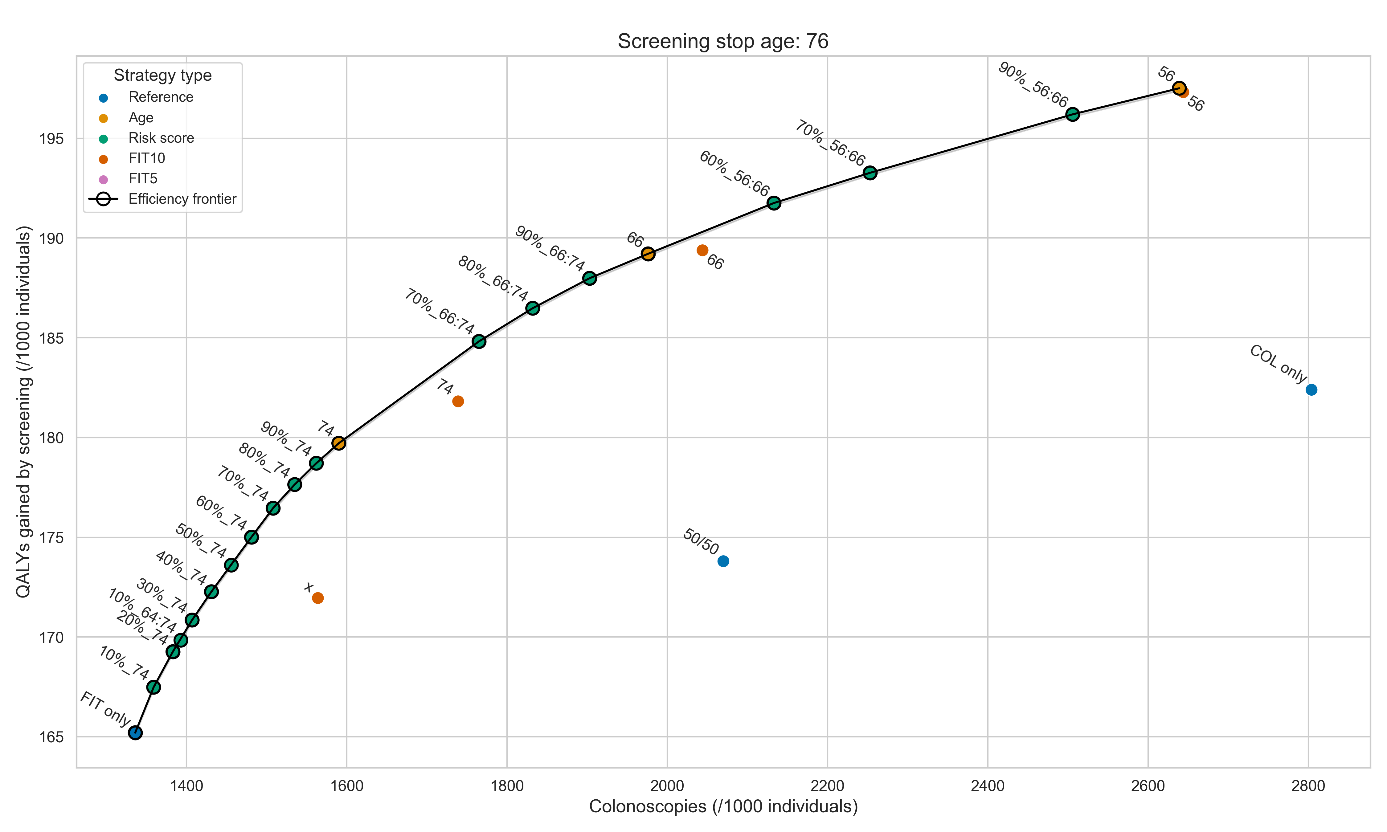
*Figure S5.5 – Overall efficient strategies in the sensitivity analysis with a screening stop age of 76.*


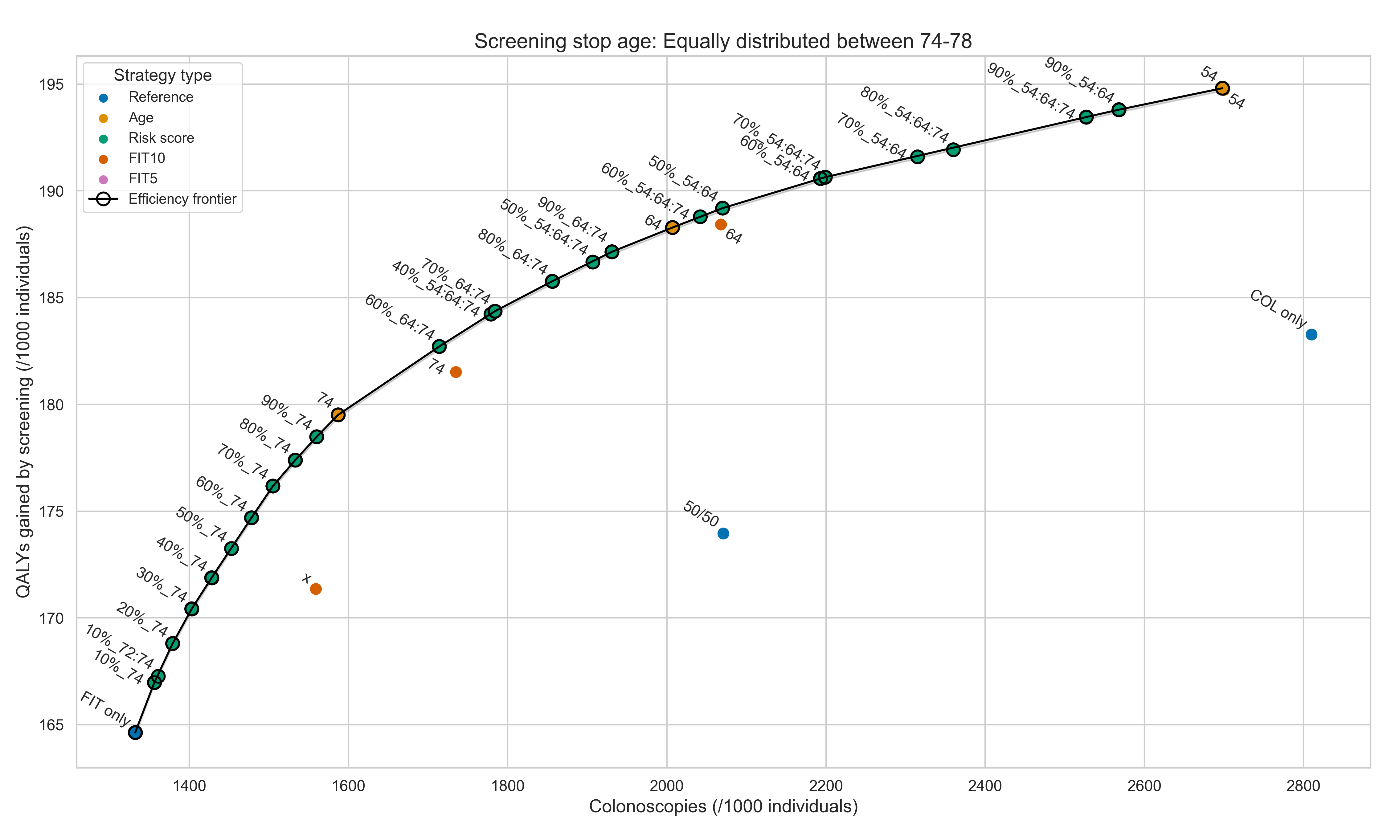
*Figure S5.6 – Overall efficient strategies in case of delayed screening cessation (33% stop at ages 74, 76 and 78, respectively).*


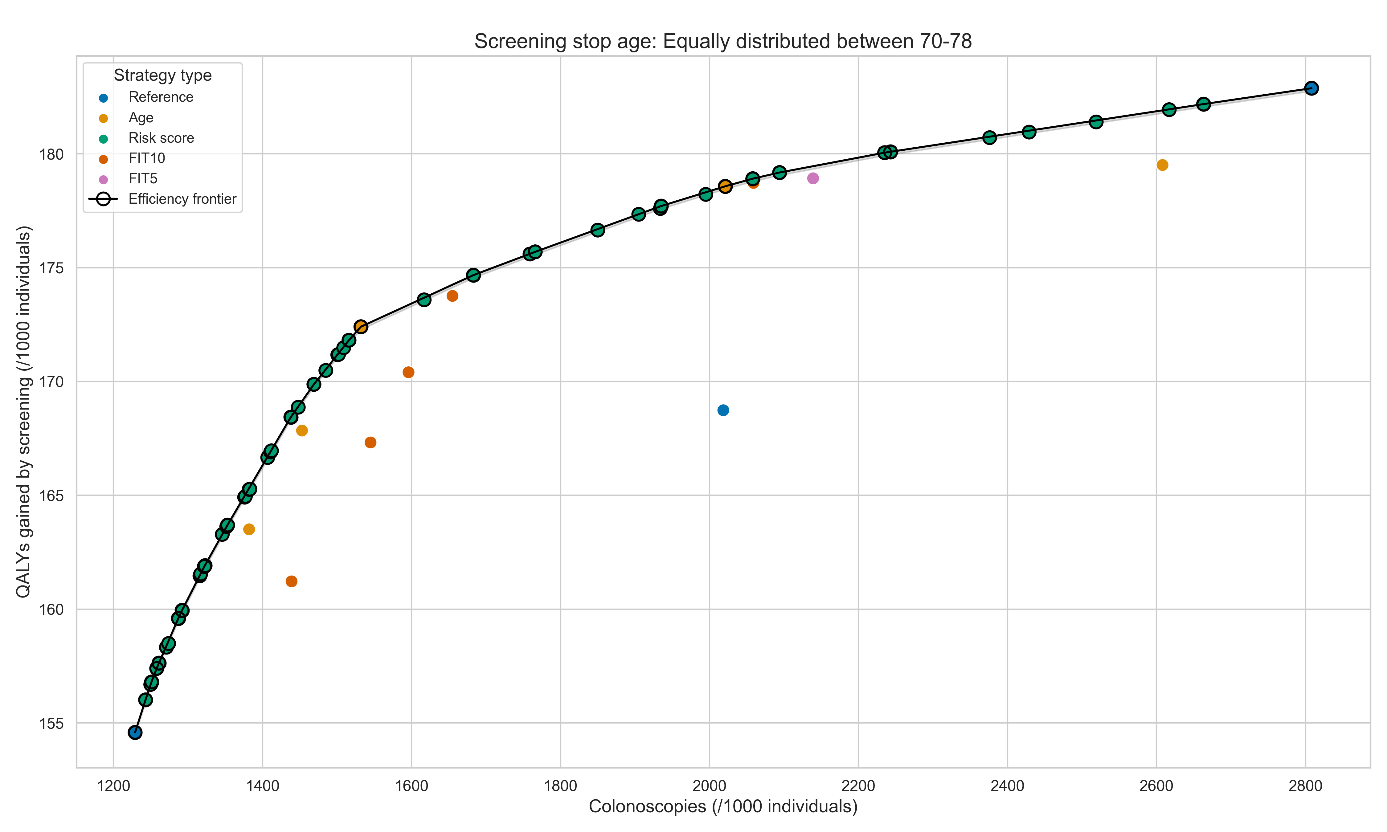
*Figure S5.7 – Overall efficient strategies in case of early and delayed screening cessation (20% stop at ages 70, 72, 74, 76 and 78, respectively). Labels are omitted due to a lack of space. Outcomes per strategy are shown in Table S5.2.*


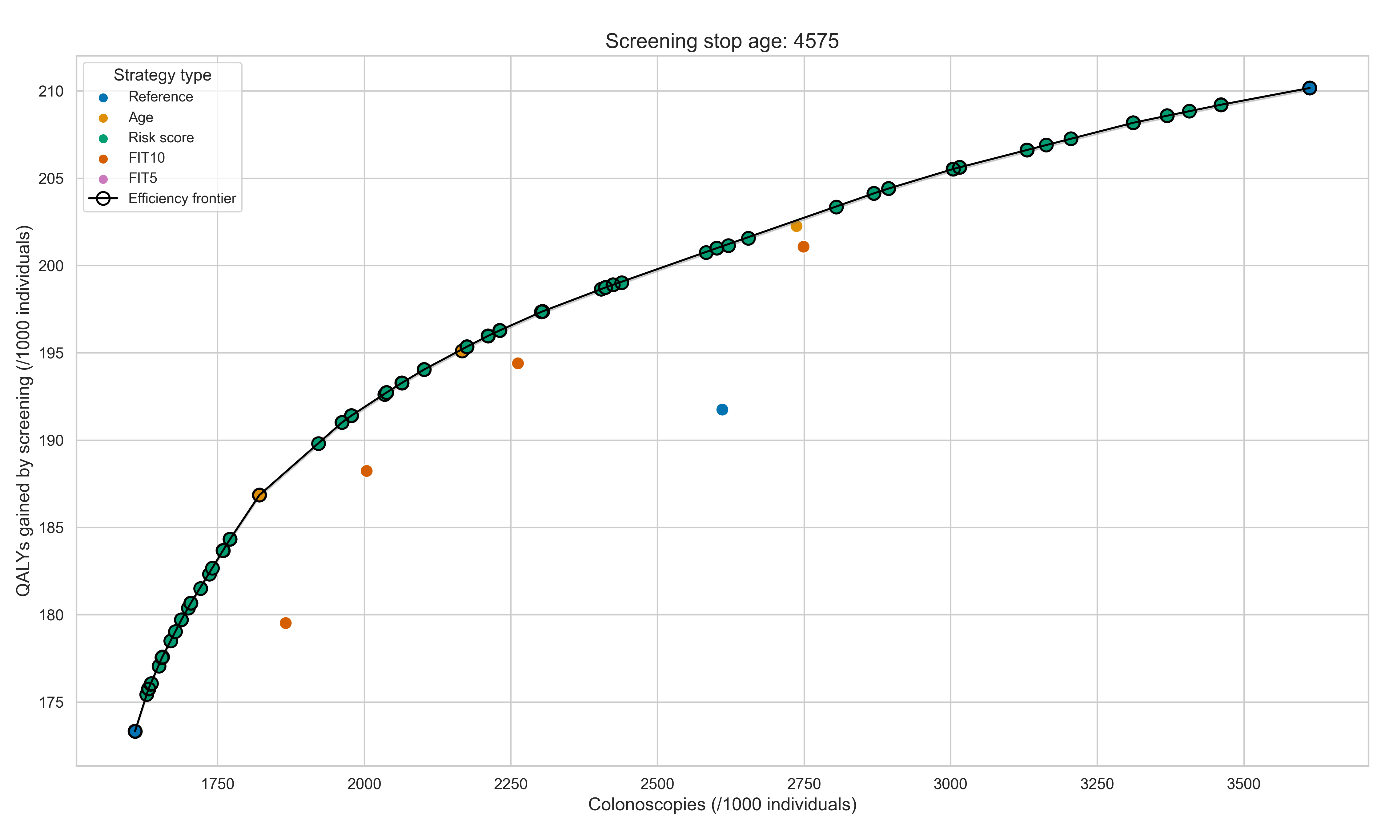
*Figure S5.8 - Overall efficient strategies when screening is recommended between ages 45 and 75, in line with USPSTF recommendations. Labels are omitted due to a lack of space. Outcomes are shown in Table S5.3.*

*Table S5.1 - Outcomes of the efficient strategies within each strategy type in the sensitivity analysis which discounted QALYs gained by 3% annually. The last column indicates which strategies are overall efficient, i.e. efficient when considering all age-based, risk-score-based, and FIT-based strategies. This table corresponds with Figure S5.2.*

| **Strategy type** | **Age-based switch criteria** | **Risk-score- or**  **FIT-based switch criteria** | **Number of colonoscopies (/1000 individuals)** | **QALYs gained**  **(/1000 individuals)** | **Overall efficient strategy** |
| --- | --- | --- | --- | --- | --- |
| Reference | FIT only |  | 1243 | 66.22 | Yes |
| Risk score | 74 | 10% | 1268 | 67.25 | Yes |
| Risk score | 72, 74 | 10% | 1274 | 67.41 | No |
| Risk score | 70, 74 | 10% | 1280 | 67.54 | No |
| Risk score | 70, 72, 74 | 10% | 1281 | 67.56 | No |
| Risk score | 68, 74 | 10% | 1289 | 67.77 | No |
| Risk score | 74 | 20% | 1294 | 68.04 | Yes |
| Risk score | 64, 74 | 10% | 1305 | 68.31 | No |
| Risk score | 74 | 30% | 1320 | 68.74 | Yes |
| Risk score | 72, 74 | 30% | 1335 | 68.99 | No |
| Risk score | 74 | 40% | 1346 | 69.36 | Yes |
| Risk score | 54, 64, 74 | 10% | 1352 | 69.41 | No |
| Risk score | 72, 74 | 40% | 1364 | 69.62 | No |
| Risk score | 64, 74 | 20% | 1370 | 69.8 | No |
| Risk score | 74 | 50% | 1373 | 69.94 | No |
| Risk score | 74 | 60% | 1401 | 70.54 | Yes |
| Risk score | 74 | 70% | 1430 | 71.15 | Yes |
| FIT10 | x | 10μg/g | 1458 | 68.96 | No |
| Risk score | 74 | 80% | 1460 | 71.65 | Yes |
| Risk score | 74 | 90% | 1489 | 72.09 | Yes |
| Age | 74 |  | 1519 | 72.5 | Yes |
| Risk score | 72, 74 | 90% | 1530 | 72.52 | No |
| Risk score | 64, 74 | 50% | 1582 | 73.2 | No |
| Risk score | 54, 64, 74 | 30% | 1595 | 73.37 | No |
| Risk score | 64, 74 | 60% | 1654 | 74.16 | Yes |
| FIT10 | 74 | 10μg/g | 1654 | 73.37 | No |
| Risk score | 54, 64, 74 | 40% | 1722 | 74.87 | Yes |
| Risk score | 64, 74 | 70% | 1728 | 74.93 | Yes |
| Risk score | 64, 74 | 80% | 1804 | 75.59 | Yes |
| Risk score | 54, 64, 74 | 50% | 1859 | 76.07 | Yes |
| Risk score | 64, 74 | 90% | 1884 | 76.23 | No |
| Age | 64 |  | 1965 | 76.74 | No |
| Risk score | 54, 64, 74 | 60% | 2001 | 77.09 | Yes |
| FIT10 | 64 | 10μg/g | 2016 | 76.8 | No |
| Reference | 50/50 |  | 2023 | 71.64 | No |
| Risk score | 54, 64 | 50% | 2032 | 77.31 | Yes |
| Risk score | 54, 64 | 60% | 2161 | 78.15 | Yes |
| Risk score | 54, 64, 74 | 70% | 2169 | 78.19 | Yes |
| Risk score | 54, 64 | 70% | 2290 | 78.78 | Yes |
| Risk score | 54, 64, 74 | 80% | 2339 | 78.98 | No |
| Risk score | 54, 64 | 80% | 2421 | 79.36 | No |
| Risk score | 54, 64, 74 | 90% | 2515 | 79.86 | Yes |
| Risk score | 54, 64 | 90% | 2559 | 80.05 | Yes |
| Age | 54 |  | 2696 | 80.61 | No |
| FIT10 | 54 | 10μg/g | 2698 | 80.63 | Yes |
| Reference | COL only |  | 2804 | 77.05 | No |

*Table S5.2 – Outcomes of the efficient strategies within each strategy type in the sensitivity analysis assuming early and delayed screening cessation (20% of individuals stops at ages 70, 72, 74, 76 and 78, respectively). The last column indicates which strategies are overall efficient, i.e. efficient when considering all age-based, risk-score-based, and FIT-based strategies. This table corresponds with Figure S5.7*

| **Strategy type** | **Age-based switch criteria** | **Risk-score- or**  **FIT-based switch criteria** | **Number of colonoscopies (/1000 individuals)** | **QALYs gained**  **(/1000 individuals)** | **Overall efficient strategy** |
| --- | --- | --- | --- | --- | --- |
| Reference | FIT only |  | 1229 | 154.59 | Yes |
| Risk score | 74 | 10% | 1243 | 156.02 | Yes |
| Risk score | 72 | 10% | 1250 | 156.69 | No |
| Risk score | 72, 74 | 10% | 1251 | 156.8 | Yes |
| Risk score | 70 | 10% | 1258 | 157.4 | Yes |
| Risk score | 70, 72 | 10% | 1261 | 157.63 | Yes |
| Risk score | 72 | 20% | 1271 | 158.33 | No |
| Risk score | 72, 74 | 20% | 1274 | 158.49 | No |
| Risk score | 70 | 20% | 1287 | 159.6 | Yes |
| Risk score | 70, 72 | 20% | 1292 | 159.95 | Yes |
| Risk score | 70, 74 | 30% | 1316 | 161.46 | No |
| Risk score | 70 | 30% | 1317 | 161.54 | No |
| Risk score | 70, 72, 74 | 30% | 1322 | 161.88 | Yes |
| Risk score | 70, 72 | 30% | 1323 | 161.91 | No |
| Risk score | 70 | 40% | 1346 | 163.28 | No |
| Risk score | 70, 72, 74 | 40% | 1352 | 163.63 | No |
| Risk score | 70, 72 | 40% | 1353 | 163.69 | Yes |
| Risk score | 70 | 50% | 1376 | 164.92 | No |
| Risk score | 70, 74 | 50% | 1377 | 164.96 | No |
| Risk score | 70, 72, 74 | 50% | 1382 | 165.26 | No |
| Age | 74 |  | 1382 | 163.51 | No |
| Risk score | 70, 72 | 50% | 1383 | 165.27 | No |
| Risk score | 70 | 60% | 1407 | 166.66 | No |
| Risk score | 70, 72 | 60% | 1411 | 166.91 | No |
| Risk score | 70, 72, 74 | 60% | 1412 | 166.95 | No |
| Risk score | 70 | 70% | 1438 | 168.44 | Yes |
| FIT10 | x | 10μg/g | 1439 | 161.22 | No |
| Risk score | 70, 72 | 70% | 1448 | 168.87 | No |
| Age | 72 |  | 1453 | 167.84 | No |
| Risk score | 70 | 80% | 1469 | 169.88 | Yes |
| Risk score | 70, 72 | 80% | 1485 | 170.48 | No |
| Risk score | 70 | 90% | 1501 | 171.17 | No |
| Risk score | 70, 74 | 90% | 1502 | 171.19 | No |
| Risk score | 70, 72, 74 | 90% | 1509 | 171.48 | No |
| Risk score | 70, 72 | 90% | 1516 | 171.81 | Yes |
| Age | 70 |  | 1532 | 172.4 | Yes |
| FIT10 | 74 | 10μg/g | 1545 | 167.32 | No |
| FIT10 | 72 | 10μg/g | 1596 | 170.4 | No |
| Risk score | 50, 60, 70 | 30% | 1617 | 173.59 | No |
| FIT10 | 70 | 10μg/g | 1655 | 173.76 | No |
| Risk score | 60, 70 | 60% | 1683 | 174.67 | Yes |
| Risk score | 50, 60, 70 | 40% | 1759 | 175.6 | No |
| Risk score | 60, 70 | 70% | 1766 | 175.69 | Yes |
| Risk score | 60, 70 | 80% | 1850 | 176.65 | No |
| Risk score | 50, 60, 70 | 50% | 1905 | 177.34 | No |
| Risk score | 60, 68 | 90% | 1934 | 177.6 | No |
| Risk score | 60, 70 | 90% | 1935 | 177.71 | Yes |
| Risk score | 60, 62 | 90% | 1995 | 178.22 | No |
| Reference | 50/50 |  | 2018 | 168.73 | No |
| Age | 60 |  | 2021 | 178.57 | Yes |
| Risk score | 50, 60, 70 | 60% | 2058 | 178.91 | Yes |
| FIT10 | 60 | 10μg/g | 2059 | 178.72 | No |
| Risk score | 50, 60 | 50% | 2094 | 179.18 | Yes |
| FIT5 | 60 | 5μg/g | 2139 | 178.92 | No |
| Risk score | 50, 60 | 60% | 2235 | 180.05 | Yes |
| Risk score | 50, 60, 70 | 70% | 2243 | 180.09 | No |
| Risk score | 50, 60 | 70% | 2376 | 180.71 | No |
| Risk score | 50, 60, 70 | 80% | 2429 | 180.95 | No |
| Risk score | 50, 60 | 80% | 2519 | 181.4 | No |
| Age | 52 |  | 2608 | 179.51 | No |
| Risk score | 50, 60, 70 | 90% | 2617 | 181.94 | No |
| Risk score | 50, 60 | 90% | 2663 | 182.18 | Yes |
| Reference | COL only |  | 2808 | 182.88 | Yes |

*Table S5.3 - Outcomes of the efficient strategies within each strategy type in the sensitivity analysis which assumed screening between ages 45 and 75, in line with USPSTF guidelines. The last column indicates which strategies are overall efficient, i.e. efficient when considering all age-based, risk-score-based, and FIT-based strategies. This table corresponds with Figure S5.8.*

| **Strategy type** | **Age-based switch criteria** | **Risk-score- or**  **FIT-based switch criteria** | **Number of colonoscopies (/1000 individuals)** | **QALYs gained**  **(/1000 individuals)** | **Overall efficient strategy** |
| --- | --- | --- | --- | --- | --- |
| Reference | FIT only |  | 1609 | 173.33 | Yes |
| Risk score | 73 | 10% | 1629 | 175.43 | No |
| Risk score | 73, 75 | 10% | 1632 | 175.76 | Yes |
| Risk score | 71, 75 | 10% | 1636 | 176.02 | No |
| Risk score | 71, 73, 75 | 10% | 1637 | 176.05 | No |
| Risk score | 73 | 20% | 1650 | 177.05 | No |
| Risk score | 73, 75 | 20% | 1655 | 177.55 | Yes |
| Risk score | 65, 75 | 10% | 1656 | 177.58 | No |
| Risk score | 73 | 30% | 1670 | 178.5 | No |
| Risk score | 73, 75 | 30% | 1678 | 179.03 | No |
| Risk score | 55, 65, 75 | 10% | 1688 | 179.71 | Yes |
| Risk score | 73, 75 | 40% | 1700 | 180.38 | No |
| Risk score | 65, 75 | 20% | 1704 | 180.67 | Yes |
| Risk score | 73, 75 | 50% | 1721 | 181.5 | No |
| Risk score | 73 | 60% | 1736 | 182.32 | No |
| Risk score | 73, 75 | 60% | 1741 | 182.66 | No |
| Risk score | 73 | 70% | 1759 | 183.68 | Yes |
| Risk score | 73, 75 | 70% | 1771 | 184.33 | Yes |
| Age | 75 |  | 1821 | 186.85 | Yes |
| FIT10 | x | 10μg/g | 1866 | 179.52 | No |
| Risk score | 65, 75 | 60% | 1922 | 189.8 | No |
| Risk score | 55, 65, 75 | 40% | 1962 | 191.01 | Yes |
| Risk score | 65, 75 | 70% | 1978 | 191.41 | Yes |
| FIT10 | 75 | 10μg/g | 2004 | 188.24 | No |
| Risk score | 45, 55, 65, 75 | 30% | 2035 | 192.61 | No |
| Risk score | 65, 75 | 80% | 2038 | 192.73 | Yes |
| Risk score | 55, 65, 75 | 50% | 2064 | 193.28 | Yes |
| Risk score | 65, 75 | 90% | 2102 | 194.05 | Yes |
| Age | 65 |  | 2167 | 195.1 | No |
| Risk score | 55, 65, 75 | 60% | 2175 | 195.35 | Yes |
| Risk score | 45, 55, 65, 75 | 40% | 2211 | 195.97 | Yes |
| Risk score | 45, 55, 65 | 30% | 2231 | 196.29 | Yes |
| FIT10 | 65 | 10μg/g | 2262 | 194.4 | No |
| Risk score | 55, 65 | 60% | 2302 | 197.35 | Yes |
| Risk score | 55, 65, 75 | 70% | 2304 | 197.38 | Yes |
| Risk score | 55, 65 | 70% | 2404 | 198.64 | No |
| Risk score | 45, 55, 65 | 40% | 2411 | 198.74 | Yes |
| Risk score | 45, 55, 65, 75 | 50% | 2424 | 198.9 | Yes |
| Risk score | 55, 65, 75 | 80% | 2439 | 199.01 | No |
| Risk score | 55, 65, 75 | 90% | 2583 | 200.74 | No |
| Risk score | 45, 55, 65 | 50% | 2601 | 201 | Yes |
| Reference | 50/50 |  | 2610 | 191.75 | No |
| Risk score | 55, 65 | 90% | 2621 | 201.14 | No |
| Risk score | 45, 55, 65, 75 | 60% | 2655 | 201.56 | No |
| Age | 55 |  | 2737 | 202.26 | No |
| FIT10 | 55 | 10μg/g | 2749 | 201.08 | No |
| Risk score | 45, 55, 65 | 60% | 2805 | 203.35 | No |
| Risk score | 45, 55 | 50% | 2869 | 204.14 | Yes |
| Risk score | 45, 55, 65, 75 | 70% | 2894 | 204.41 | Yes |
| Risk score | 45, 55, 65 | 70% | 3004 | 205.53 | Yes |
| Risk score | 45, 55 | 60% | 3015 | 205.63 | Yes |
| Risk score | 45, 55, 65, 75 | 80% | 3130 | 206.62 | Yes |
| Risk score | 45, 55 | 70% | 3163 | 206.9 | No |
| Risk score | 45, 55, 65 | 80% | 3205 | 207.26 | No |
| Risk score | 45, 55 | 80% | 3311 | 208.18 | Yes |
| Risk score | 45, 55, 65, 75 | 90% | 3369 | 208.58 | Yes |
| Risk score | 45, 55, 65 | 90% | 3407 | 208.84 | No |
| Risk score | 45, 55 | 90% | 3461 | 209.21 | Yes |
| Reference | COL only |  | 3612 | 210.17 | Yes |

References

1. Loeve F, Boer R, Van Oortmarssen GJ, et al. The MISCAN-COLON simulation model for the evaluation of colorectal cancer screening. *Computers and Biomedical Research* 1999;32(1):13-33.

2. Van Hees F, Zauber AG, Van Veldhuizen H, et al. The value of models in informing resource allocation in colorectal cancer screening: the case of the Netherlands. *Gut* 2015;64(12):1985-97.

3. Gini A, Buskermolen M, Senore C, et al. Development and validation of three regional microsimulation models for predicting colorectal cancer screening benefits in Europe. *MDM Policy & Practice* 2021;6(1):2381468320984974.

4. Fortin F-A, De Rainville F-M, Gardner M-AG, et al. DEAP: Evolutionary algorithms made easy. *The Journal of Machine Learning Research* 2012;13(1):2171-75.

5. Faivre J, Bossard N, Jooste V. Trends in net survival from colon cancer in six European Latin countries. *European Journal of Cancer Prevention* 2017;26:40-47.

6. Lepage C, Bossard N, Dejardin O, et al. Trends in net survival from rectal cancer in six European Latin countries. *European Journal of Cancer Prevention* 2017;26:48-55.

7. Lutz JM, Pury P, Fioretta G, Raymond L. The impact of coding process on observed cancer mortality trends in Switzerland. *European Journal of Cancer Prevention* 2004:77-81.

8. Enquête suisse sur la santé: Office fédéral de la statistique; [Available from: <https://www.bfs.admin.ch/bfs/fr/home/statistiques/sante/enquetes/sgb.html>.

9. Van Duuren LA, Bulliard J-L, Mohr E, et al. Population-level impact of the BMJ Rapid Recommendation for colorectal cancer screening: a microsimulation analysis. *BMJ Open Gastroenterology* 2024;11(1):e001344. doi: 10.1136/bmjgast-2023-001344

10. Hippisley-Cox J, Coupland C. Development and validation of risk prediction algorithms to estimate future risk of common cancers in men and women: prospective cohort study. *BMJ open* 2015;5(3):e007825.

11. QCancer®(15yr,colorectal) risk calculator [updated 15th March 2019. Available from: <https://qcancer.org/15yr/colorectal/index.php> accessed 16th June 2023.

12. Peterse EFP, Meester RGS, de Jonge L, et al. Comparing the cost-effectiveness of innovative colorectal cancer screening tests. *JNCI: Journal of the National Cancer Institute* 2021;113(2):154-61.

13. Harlass M, Knudsen AB, Nieboer D, et al. Benefits of colorectal cancer screening using FIT with varying positivity thresholds by age and sex. *JNCI: Journal of the National Cancer Institute* 2025:djaf149.

14. Van Rijn JC, Reitsma JB, Stoker J, et al. Polyp miss rate determined by tandem colonoscopy: a systematic review. *Official journal of the American College of Gastroenterology| ACG* 2006;101(2):343-50.

15. Brändle K, Bulliard J-L. Premier bilan du programme vaudois de dépistage du cancer colorectal. *Rev Medical Suisse* 2022;18(803):2108-11. doi: 10.53738/REVMED.2022.18.803.2108
